# Modulation of Rab7a-mediated growth factor receptor trafficking inhibits islet beta cell apoptosis and autophagy under conditions of metabolic stress

**DOI:** 10.1038/s41598-020-72939-y

**Published:** 2020-09-25

**Authors:** Nirun V. Hewawasam, Fadel Lhaf, Henry A. Taylor, Katrina Viloria, Amazon Austin, Aileen King, Peter Jones, Lucy Jones, Mark D. Turner, Natasha J. Hill

**Affiliations:** 1 Department of Biomolecular Sciences, Kingston University, Penrhyn Road, Kingston-upon-Thames, KT1 2EE UK; 2 Department of Diabetes, Kings College London, London, UK; 3 School of Science and Technology, Nottingham Trent University, Nottingham, UK

**Keywords:** Cell biology, Endocrine system and metabolic diseases, Metabolic disorders

## Abstract

Regenerative medicine approaches to enhancing beta cell growth and survival represent potential treatments for diabetes. It is known that growth factors such as insulin, IGF-1 and HGF support beta cell growth and survival, but in people with type 2 diabetes the destructive effects of metabolic stress predominate and beta cell death or dysfunction occurs. In this study we explore the novel hypothesis that regulation of growth factor receptor trafficking can be used to promote islet beta cell survival. Growth factor signalling is dependent on the presence of cell surface receptors. Endosomal trafficking and subsequent recycling or degradation of these receptors is controlled by the Rab GTPase family of proteins. We show that Rab7a siRNA inhibition enhances IGF-1 and HGF signalling in beta cells and increases expression of the growth factor receptors IGF-1R and c-Met. Furthermore, Rab7a inhibition promotes beta cell growth and islet survival, and protects against activation of apoptosis and autophagy pathways under conditions of metabolic stress. This study therefore demonstrates that Rab7a-mediated trafficking of growth factor receptors controls beta cell survival. Pharmaceutical Rab7a inhibition may provide a means to promote beta cell survival in the context of metabolic stress and prevent the onset of type 2 diabetes.

## Introduction

There are currently 382 million people affected by diabetes, with this figure estimated to reach 592 million by 2035^1^. Loss or insufficiency of insulin producing beta cells is a causative factor in both type 1 and type 2 diabetes. In type 1 diabetes beta cells are destroyed by autoreactive immune cells, while in type 2 diabetes the number or function of beta cells is compromised and insulin production is insufficient to cope with demand^2,3^. In type 2 diabetes, the inflammatory and metabolic conditions, including high circulating levels of saturated fatty acids, place a particular stress on beta cells that contributes to their demise^4^. Therefore, regenerative medicine approaches to enhancing beta cell growth and survival represent potential treatments for diabetes.

There are many factors known to regulate islet growth in the adult pancreas, including growth factors, amino acids, glucose, insulin and gestational hormones^5–8^. Both insulin-like growth factor-1 (IGF-1) and hepatocyte growth factor (HGF) have been shown to increase islet beta cell proliferation in vivo and in vitro^9–12^. Loss of HGF signalling in pancreatic beta cells inhibits beta cell survival and accelerates the onset of diabetes, and can similarly cause gestational diabetes and incomplete maternal beta cell adaptation^10,13^. There is a complex cross-talk between IGF-1, insulin and the IGF-1 and insulin receptors that is challenging to unravel in vivo, but it is clear that the combined loss of insulin and IGF-1 receptors in beta cells results in diabetes^14^. In terms of intracellular signalling, HGF signals through the cMET receptor to activate multiple signalling pathways including Ras-ERK and P13K/Akt^15^. IGF-1 binding to IGF-1R similarly signals through multiple pathways including PI3K-mammalian target of rapamycin (mTOR), as well as activating Ras-ERK^16^. However, although much is now known about the role of growth factors in regulating islet beta cell proliferation, it has not yet proved possible to sufficiently promote islet expansion for therapeutic use.

Growth factor responses are dependent upon the presence of growth factor receptors at the cell surface. Cell surface receptor density can be increased by de novo synthesis of receptors, by recycling from intracellular stores or by preventing receptor internalisation. Growth factor receptors can also activate distinct signalling pathways depending on their localization at either the cell surface or in endosomes^17^. The regulation of growth factor receptor trafficking is therefore critical in the regulation of cellular growth and survival.

Rab GTPases mediate vesicular transport events and play a primary role in the regulation of receptor trafficking. Rab proteins regulate both biosynthetic and endocytic pathway trafficking, including the degradation and recycling of surface receptors, and thus regulate cell growth and differentiation^18^. Following initial endocytosis, cell surface receptors in early endosomal vesicles are sorted into divergent pathways, entering either a fast recycling pathway dependent on Rab4, a slow recycling pathway dependent on Rab11, or the lysosomal degradation pathway dependent on Rab7a^19^. In haematopoetic cells, inhibiting Rab7a prevents trafficking of nutrient transporter proteins to the lysosome following growth factor withdrawal. Instead, nutrient receptor expression at the cell surface is maintained, promoting cell survival^20^. Rab7a also regulates trafficking of EGFR, the NGF receptor Trk1, and the angiogenic receptor Nrp1^21–23^, and is essential for the process of autophagy^24,25^. Together, these studies suggest the possibility that Rab7a inhibition could be used therapeutically to promote growth factor responsiveness.

Here, we test the hypothesis that Rab7a inhibition can be used to increase growth factor receptor density in beta cells in order to enhance growth factor responses, and that this could be used to promote beta cell survival for the treatment of diabetes.

## Materials and methods

### Islet isolation and culture

Pancreatic islets were isolated from ICR mice aged 8–12 weeks (Harlan, Huntingdon UK). All experimental protocols were approved by the animal welfare and ethical review board at King’s College London and in accordance with the UK. Home Office Animals (Scientific Procedures) Act 1986 with 2012 amendments (establishment licence: X24D82DFF; project licence: PBCFBE464). Islets were isolated using collagenase digestion and separated by density gradient centrifugation as previously described^26^. After washing, islets were handpicked and cultured overnight in RPMI medium containing 11.1 mM d-glucose and additionally supplemented with 10% foetal bovine serum (FBS), Penicillin (100 units/ml) and streptomycin (100 µg/ml).

### Cell culture

All cell culture reagents were purchased from Gibco Life Technologies unless otherwise stated. Insulinoma-1 (INS-1) cells were obtained from Dr E.C. Edling, Queen Mary University of London, and grown in Roswell Park Memorial Institute (RPMI) 1640 medium containing 11.1 mM d-glucose and 2.1 mM l-glutamine supplemented with 10% heat-inactivated FBS, 2 mM l-glutamine, 10 mM HEPES (pH 7.2–7.5), 1 mM Sodium Pyruvate, Penicillin (100 units/ml), streptomycin (100 µg/ml) and 0.05 mM 2-mercaptoethanol (Sigma). Cells were grown in T75 flasks (Nunc/Fisher Thermo Scientific) at 37 °C under 5% CO_2_ and passaged every 3–4 days using 0.05% Trypsin EDTA. Experiments were performed within ten passages. Insulin expression by imunocytochemistry was used to authenticate beta cell status. Mycoplasma status has not been tested.

For experiments involving growth factor treatment, lyophilised IGF-1 or HGF (R&D Systems) was re-suspended according to the manufacturer’s instructions at 100 μg/ml in sterile PBS. Following Rab7a knockdown, INS-1 cells (1 × 10^5^ cells/well in 48 well plate) and islet cells were starved overnight in low serum medium (0.5% or 0% FBS as indicated). For islets, 60 islets per treatment were typically used, and islets were dispersed into single cells using Accutase treatment. The cells were then stimulated with either IGF-1 or HGF (20 or 200 ng/ml as indicated) in low serum medium for between 0 and 60 min.

### Immunohistochemistry

Whole mouse pancreas was removed, fixed in 10% Neutral Buffered Formalin (NBF) (pH 6.8–7.2) for 24 h and embedded in paraffin. Sections (5 μm) were de-paraffinised using Histoclear (AGTC Bioproducts), incubated with PBS and 0.3% Triton X-100 (Sigma) and blocked with 10% normal horse serum (Sigma). For Rab7a staining, tissue sections were incubated overnight at 4 °C with Rab7a antibody (Abcam ab137029, diluted 1/25–1/100 in 10% normal horse serum. The following day slides were incubated with goat anti-rabbit biotinylated secondary antibody (Vector Laboratories-BA1000; diluted 1/250) for 90 min at ambient temperature. Non-specific activity of endogenous peroxidase activity was minimised by pre-treatment of slides with 1% hydrogen peroxide for 15 min. For visualisation, the ABC/DAB system was used according to the manufacturer’s instructions (Vector Laboratories). Sections were counter stained with haematoxylin and visualised using a Nikon Eclipse 80i microscope and NIS element software.

### Fluorescence immunocytochemistry

For Rab7a staining, INS-1 cells (2 × 10^5^ cells/well) were grown on poly-lysine coated coverslips and fixed in 10% NBF, then incubated with Rab7a primary antibody (1/1000; Abcam, ab137029) followed by secondary Alexa Fluor 488 secondary antibody (1:200; Invitrogen 10256302). SlowFade Gold Antifade DAPI (Thermo Scientific) was used to mount coverslips and visualise nuclei. Slides were viewed using a Leica TCS AOBS inverted laser scanning confocal microscope and X63 oil immersion objective. All images within a single experiment were obtained and processed under identical settings.

For growth factor receptor staining, INS-1 cells (2 × 10^5^ cells/well) were grown on poly-lysine coated coverslips in a six well plate. Following Rab7a knockdown, cells were fixed in 10% NBF and washed using cold PBS containing Ca^2+^ and Mg^2+^. To minimise non-specific binding cells were blocked using 10% normal horse serum for 1 h at room temperature followed by overnight incubation at 4 °C with IGF-1R and c-Met primary antibodies (both diluted 1:250, IGF-1R 9750S Cell Signaling Technology; c-Met SC-162R Santa Cruz Biotechnology). The next day, cells were washed and treated with Alexa Fluor 488 secondary antibody (1:350) and Cell Mask Orange (Invitrogen VXC10045) to stain the plasma cell membrane. Coverslips were mounted onto slides using SlowFade Gold Antifade with DAPI. Images were captured using a 63× oil immersion objective on a Leica TCS SP2 AOBS inverted laser scanning confocal microscope. All images in a single experiment were taken and processed using identical settings. Quantification of receptor expression was performed using ImageJ. Binary images were created for each green channel image using a constant threshold, and the pixel number above this threshold measured for each image. To standardise for variability in the number of cells, the number of cells/image was quantified using the DAPI stain and the Analyse Particles function in ImageJ^27^.

For the Caspase-3/7 apoptosis assay, cultures were supplemented with 5 µm non-fluorescent caspase − 3/− 7 substrate (DEVD) that releases a green DNA binding fluorescent label upon caspase activation and cleavage (Essen Bioscience). Apoptotic cells therefore fluoresce. An IncuCyte Zoom Live Cell Imaging System was used for image capture and analysis.

### siRNA transfection

For siRNA knockdown, cells were treated with either Rab7a siRNA (ON-TARGETplus Rat Rab7aa siRNA J-089334-09-0010, sequence GUAAAGAGAUGAGCGUGAU; ON TARGETplus mouse Rab7a siRNA D-040859-01, sequence CAGCUGGAGAGACGAGUUU); or with a control siRNA (ON-TARGETplus non-targeting D-001810-10), all purchased from Dharmacon and re-suspended at 100 μM according to the manufacturer’s instructions. INS-1 cells were plated in six well plates at 1 × 10^5^ cells/well and immediately transfected for 3 days with the indicated concentration of siRNA using 3 μl/ml Hiperfect transfection reagent (Qiagen). For experiments using pancreatic islets, islets were allowed to recover for 24 h post isolation then washed in PBS and centrifuged at 178 × *g* (1000 rpm) for 3 min. After discarding the supernatant, the islets were incubated in a gentle dissociation solution containing 0.02% EDTA in PBS, for 1.5 min at 37 °C, as previously described^28^. Islets were then washed in complete medium, centrifuged at 178Xg for 3 min and cultured in low-serum medium containing 0.5% FBS for 2 h before transfection with 150 nM Rab7a siRNA or control siRNA complexes in 3 μl/ml Hiperfect transfection reagent. Islets were transfected for 3 days then used for downstream experiments and to confirm Rab7a knockdown by Western blot. For all experiments in this manuscript Rab7a knockdown was at least 70% compared to control siRNA treated cells except where otherwise indicated.

### Western Blot

Cells were washed with ice cold PBS and lysed with RIPA buffer (Pierce/Thermo Fisher Scientific 89900). SDS sample buffer was added to each sample (1:5 dilution) prior to boiling at 100 °C for 6 min. For INS-1 cells, typically 10 µg protein was loaded per well, and for islets the entire lysate from 60 dispersed islets was used. Lysates were resolved by SDS-PAGE using 13% gel and the proteins were electro-transferred onto nitrocellulose membranes (GE Healthcare). Intact membranes were blocked in 5% non-fat milk followed by overnight incubation at 4 °C with primary antibodies. The following primary antibodies were used in this study: Rab7a (1:1000; Abcam ab137029 or Cell Signaling Technology 9367), β-actin (1:4000; Abcam ab8224), β-tubulin (1:1000; Abcam ab6046 or Cell Signaling Technology 2128), p-ERK 1/2 (1:1000; Cell Signalling 9101S), rabbit anti-Met (Santa Cruz SP260), rabbit anti-LC3-A/B (Cell Signalling Technology 4108) and rabbit anti-Caspase-3 (Cell Signalling Technology 9662). The following day membranes were incubated with horseradish peroxidase-conjugated anti-rabbit secondary antibody (1:2500; Cell Signaling 7074). Proteins were visualised using Supersignal West Pico chemiluminescent substrate (Thermo Fisher Scientific) and a Syngene GeneGnome imaging system with GeneSnap software. Band intensities were quantified using ImageJ software^27^. Lanes have in some cases been re-ordered for clarity (indicated by line in blot). Images of the original, uncropped blots are available as “Supplementary data”, with the exception of the validation experiments in Fig. 4c,d, Supplementary Fig. 1, and for beta tubulin in Fig. 6c, where whole blot images could not be located.

### Sulphorhodamine B (SRB) assay

The SRB assay is a colourimetric assay that indirectly measures cell number by quantification of protein^29,30^. INS-1 cells treated with Rab7a or control siRNA were re-plated at 1 × 10^4^ cells/well in a 96 well plate. Cells were starved overnight in medium containing 0.5% FBS, then stimulated with 20 ng/ml IGF-1 or HGF. Some cells were treated with 20 ng/ml etoposide, or cultured in low serum (0.5% FBS) or complete (10% FBS) media. On day 5 of treatment cells were fixed using 40% w/v TCA (Thermo Scientific 10336200) at a final concentration of 10% v/v at 4 °C for 1 h and washed using water. Once sufficiently dry, proteins were stained using 0.4% w/v SRB (Sigma S1402) in 1% v/v acetic acid for 30 min at ambient temperature and washed using water. After sufficient drying 10 mM Tris at pH 10 was added to solubilise SRB and absorbance was read at 565 nm using an Epoch Microplate spectrophotometer (Bio Tek, USA).

### Live/dead assay

The LIVE/DEAD Viability/Cytotoxicity kit for mammalian cells (Thermo Scientific L-3224) was used to visualise and quantify the % live dispersed islet cells. Following Rab7a knockdown islets from 6 mice were divided equally into groups of 60 islets and incubated with Accutase (Sigma Aldrich) for 10 min at 37 °C for dispersion. Following de-activation of Accutase using complete medium, the dispersed islets were seeded onto poly-lysine coated coverslips, allowed to adhere overnight. The islet cells were then serum-starved for 12 h before treatment with serum free medium, or serum free medium containing 200 ng/ml IGF-1 and. After 2 days coverslips were stained with Calcein AM (0.25 μM) and Ethidium homodimer-1 (2.0 μM) at ambient temperature for 1 h under reduced light. Coverslips were then mounted onto glass slides and islet cells viewed using 40× and 63× oil immersion objectives on a Leica TCS SP2 AOBS inverted laser scanning confocal microscope. Six images were taken per coverslip, and two coverslips imaged per treatment in each experiment.

### Palmitic acid preparation and treatment

Palmitic acid solutions were prepared according to published protocols^31^. Briefly, 25.642 g of palmitic acid (PA) (Sigma P-500) was dissolved in 1 l of 0.1 M Sodium Hydroxide (NaOH) at 70 °C to obtain a stock 100 mM PA solution. In a 60 °C water bath, 5% (wt/vol) free fatty acid (FFA)-free bovine serum albumin (BSA) (Sigma A-6003) was prepared in serum-free RPMI medium. A stock solution of 5 mM PA/BSA complex was prepared by repeated vortexing followed by 60 °C incubation, then allowed to reach room temperature before sterile filtration (0.45 μm pore; Sigma N9020). This 5 mM PA/BSA solution was further diluted in RPMI-1640 to achieve the indicated final concentration. The solution was stored at − 20 °C for 3–4 weeks. Control cells were treated with a solution prepared identically except for the absence of PA.

### Live cell imaging

To visualise and compare the effect of palmitic acid on cell density and apoptosis, the real time live- cell imaging system IncuCyte Zoom (Essen Bioscience) was used. For these experiments INS-1 cells were transfected as above but in a 96 well format with 10,000 cells per well. The IncuCyte Caspase-3/7 reagent for apoptosis (cat. 4440) was diluted in culture medium to a final concentration of 5 µM (1:1000) and added to the wells with the palmitic acid. Data was then collected at 72 h. Confluency and number of apoptotic cells were determined using IncuCyte software (version 2015A).

### Statistical analysis

Data processing was performed as indicated in the relevant sections, and the number of replicates for each experiment is indicated in the relevant figure legend. The sample size was chosen to determine consistency of the results. Results are indicated as mean ± standard error of the mean (SEM) unless otherwise indicated. A two-tailed Students’ t-test was used for statistical analysis, and in all cases, p ≤ 0.05 was accepted as significant.

## Results

### Expression of Rab7a in pancreatic islets and beta cells

**Figure 1:**
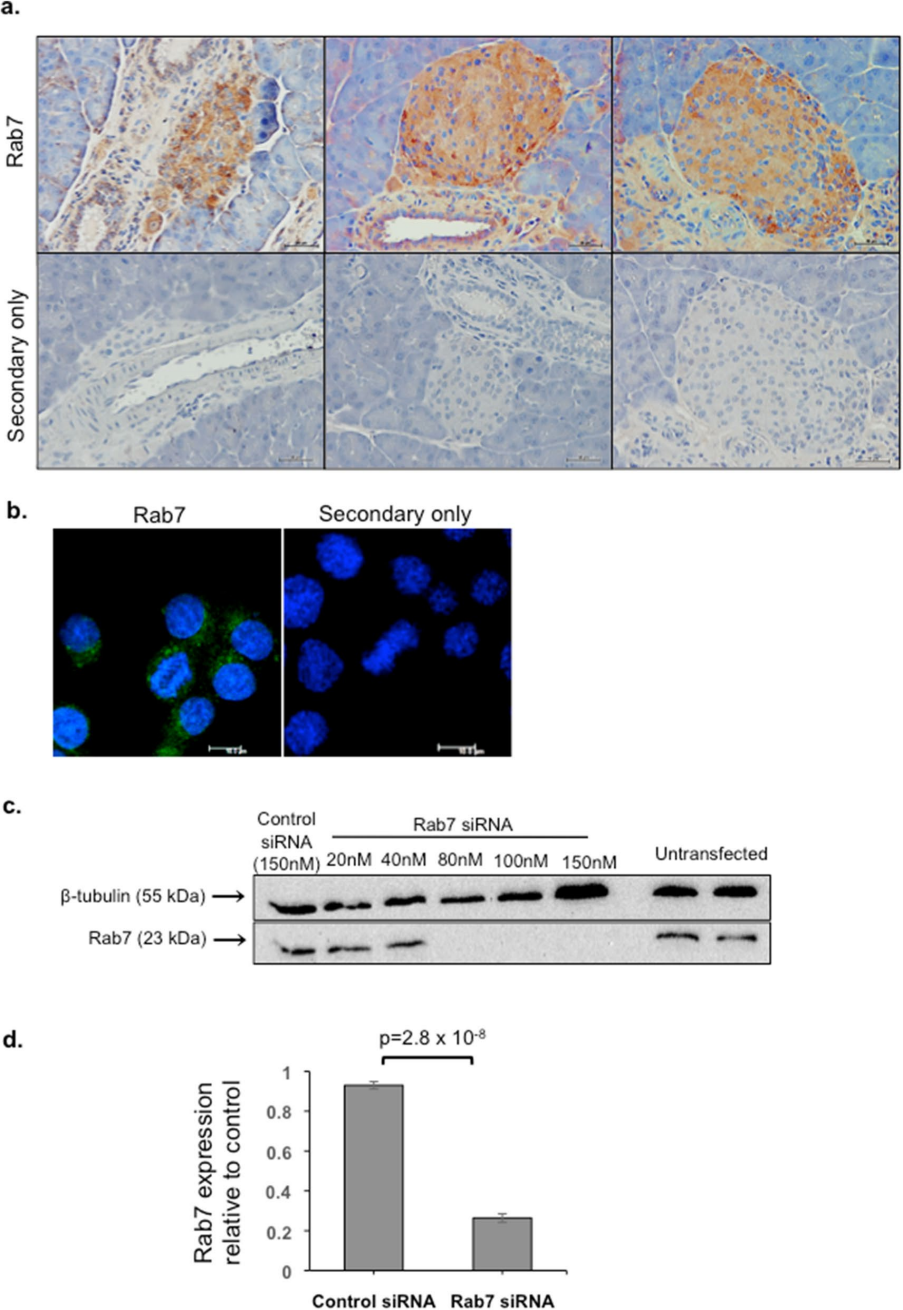
Expression of Rab7a in mouse pancreas and in beta cells. (**a**) Paraffin embedded mouse pancreas sections were immunostained using Rab7a antibody (brown; top panels) or with the secondary antibody only as a control (lower panels), and counterstained with haematoxylin. Images taken using a 20× objective and scale bar = 50 μm. Staining representative of three independent experiments. (**b**) INS-1 cells were grown on coverslips and fixed before staining with a Rab7a antibody (green) or with the Alexa 488 secondary only. Nuclei were stained with DAPI (blue). Images were taken using a 63× objective and scale bar = 10 μm. Staining is representative of multiple images taken from three slides. (**c**) INS-1 cells were treated in an initial experiment with a range of concentrations of Rab7a siRNA or negative control siRNA, or left untransfected. On day 3 of culture, cells were lysed and analysed by Western blot with antibodies to Rab7a and β tubulin as a loading control. (**d**) Quantification of Western blot detection of Rab7a expression in INS-1 cells treated with 150 nM or control or Rab7a siRNA. The graph indicates mean Rab7a expression ± SEM, n = 5 independent experiments; values are standardised to β tubulin and relative to control siRNA. Students’ t-test was used to evaluate statistical significance.

Using immunohistochemistry, we show that Rab7a is expressed throughout the pancreas including islet cells, acinar tissue, ducts and blood vessels (Fig. 1a). Fluorescence immunocytochemistry further revealed the subcellular localisation of Rab7a within the cytoplasm of INS-1 cells, with a punctate distribution consistent with the expected localisation in a late endosomal/lysosomal compartment (Fig. 1b). Rab7a expression could also be detected in INS-1 cells by western blot, and a titration of siRNA concentration showed that at least 80 nM Rab7a siRNA was required to reduce Rab7a protein expression, with maximal knockdown (98%) achieved using 150 nM siRNA (Fig. 1c). Subsequent experiments therefore utilized 150 nM siRNA and, although the extent of knockdown varied, 150 nM siRNA treatment reduced Rab7a expression by on average over 70%, as shown in Fig. 1d.

### Rab7a siRNA knockdown enhances beta cell growth

**Figure 2:**
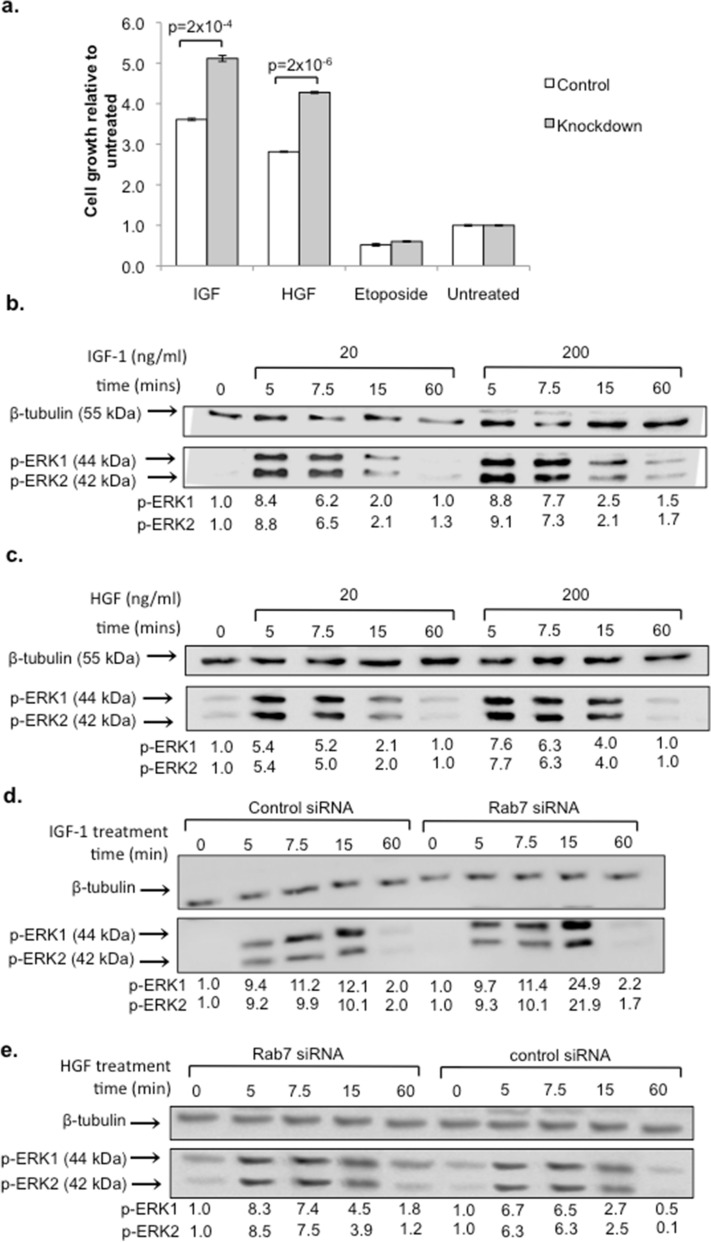
Rab7a siRNA knockdown enhances IGF-1 and HGF growth and signalling in beta cells. (**a**), INS-1 cells were transfected with 100 nM Rab7a siRNA or control siRNA for 3 days, re-plated at 1 × 10^4^ cells/well and cultured overnight in reduced serum medium. The cells were then stimulated with 20 ng/ml IGF-1, HGF or etoposide, or left untreated, and the SRB assay was performed on day 5 to determine cell growth. Graphs show mean fold change relative to control (0.5% FBS) treated cells ± SEM (n = 3; data represents three independent experiments with n = 8 replicates in each experiment). Statistical analysis in was performed using Students’ t-test. In (**b**–**e**) Western blot analysis was performed to determine activation of the signalling intermediate phospho-ERK-1/2 following overnight culture in low serum medium (0.5% FBS) and treatment with growth factor as indicated. Validation of conditions required for ERK-1/2 phosphorylation by IGF-1 (**b**) and HGF (**c**) in INS-1 cells was determined by treating with either 20 or 200 ng/ml growth factor for the indicated times (representative of two independent experiments; validation data). In (**d**, **e**), INS-1 cells were transfected with control- or Rab7a-siRNA before stimulation with 20 ng/ml IGF-1 (**d**) or HGF (**e**). Values indicate phospho-ERK 1/2 expression standardised to β-tubulin and relative to unstimulated cells. Results are representative of three independent experiments with similar timepoints.

To determine the effect of reducing Rab7a expression on beta cell growth, we investigated the effect of Rab7a siRNA knockdown in response to two important beta cell growth factors, IGF-1 and HGF. INS-1 cells were transfected with either control- or Rab7a-siRNA, re-plated at 10,000 cells/well and cultured overnight in reduced serum medium. The cells were then stimulated for 5 days with 20 ng/ml IGF-1 or HGF, with the cytotoxic compound etoposide (as a negative control), or left untreated. As shown in Fig. 2a, Rab7a siRNA transfected cells showed enhanced growth in response to both HGF and IGF-1 compared to control siRNA transfected cells. These experiments demonstrate that Rab7a inhibition enhances beta cell growth.

### Rab7a siRNA knockdown enhances growth factor signalling in beta cells

ERK 1/2 activation is one of the key signalling events involved in beta cell growth and proliferation^32–34^. In order to test whether the enhanced beta cell growth was due to increased growth factor receptor signalling, we therefore determined the effect of Rab7a siRNA treatment on ERK-1/2 activation following growth factor treatment. As shown in Fig. 2b,c, 20 ng/ml IGF-1 or HGF was sufficient to cause a 5–8-fold increase in ERK 1/2 phosphorylation, with peak activation being observed at 5 min and signal termination occurring by 60 min. We then determined the effect of Rab7a knockdown on IGF-1 and HGF induced signalling. INS-1 cells treated with Rab7a siRNA or control siRNA were re-plated on day 3 and cultured overnight in reduced serum medium. The cells were then stimulated with IGF-1 or HGF, and ERK1/2 phosphorylation was determined by Western blot. ERK1/2 phosphorylation was increased by approximately 2-fold in Rab7a knockdown cells compared to control cells after 15 min stimulation with both IGF-1 and HGF (Fig. 2d,e). This increased signalling suggests that Rab7a knockdown enhances growth factor responsiveness.

### Rab7a siRNA knockdown increases growth factor receptor density in beta cells

**Figure 3:**
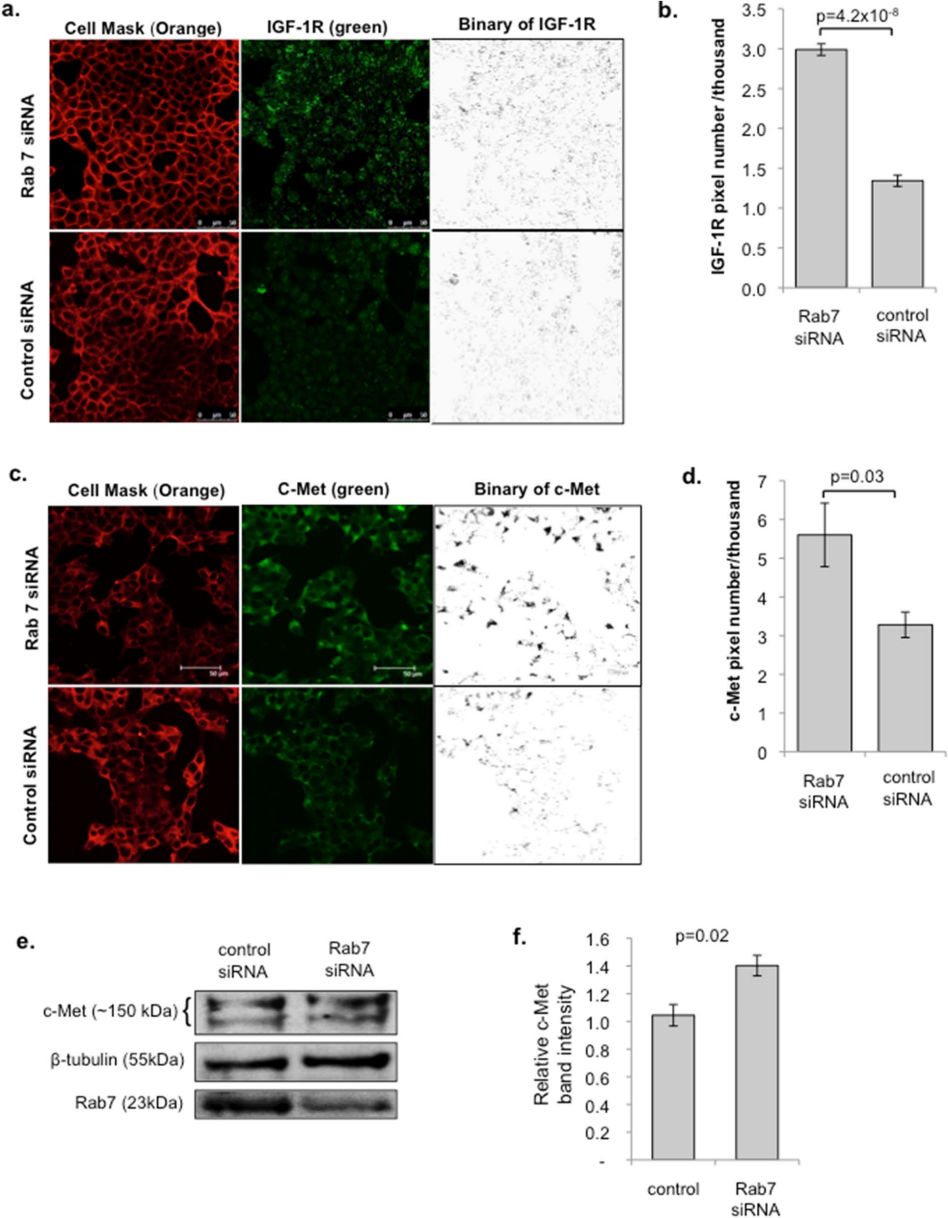
Rab7a siRNA knockdown increases growth factor receptor density in beta cells. INS-1 cells were plated on coverslips and transfected with control- or Rab7a-siRNA for 3 days. Cells were then fixed and stained with antibodies to IGF-1R (**a**, **b**) or the HGF receptor c-Met (**c**, **d**), shown in green, and with Cell Mask Orange. Representative images are shown in (**a**) and (**c**). Scale bar = 30 μm. Quantification of receptor staining was determined by calculating the green pixel number over the set threshold, as shown in the binary images, and standardised for the number of cells/image determined using DAPI staining and the cell count function in ImageJ. Graphs show mean pixel number ± SEM for IGF-1R (**b**) and c-Met (**d**). n = 8 for both IGF-1R and c-Met experiments, and data is pooled from three independent experiments. Statistical analysis was performed using Students’ t-test. Secondary only controls were negative for green fluorescent staining (data not shown). (**e**, **f**) INS-1 cells were treated with either control- or Rab7a-siRNA for 3 days, cell lysates were then prepared and analysed by western blot using the indicated antibodies. A representative blot is shown in (**e**), and mean c-Met receptor band intensity ± SEM for four independent experiments is shown in (**f**). Statistical significance was determined using Student’s paired sample t-test. Rab7a siRNA knockdown was on average ~ 50% in the experiments shown in (**e**, **f**) and therefore did not reach the threshold of 70% used in the rest of the study.

Rab7a mediates the trafficking of internalised receptors for degradation^35,36^. Having shown that Rab7a siRNA knockdown enhances growth factor receptor signalling, we therefore investigated whether this could be explained by an increase in growth factor receptor density. Rab7a siRNA and control treated cells were grown on coverslips and receptor expression was determined by quantitative confocal microscopy. As shown in Fig. 3, the expression of IGF-1R and the HGF receptor c-Met was significantly increased in Rab7a siRNA treated cells compared to control siRNA treated cells. This difference is clearly represented in the binary images of the green channel after thresholding, as well in the quantified data. The increased expression of c-Met following Rab7a siRNA knockdown was further confirmed by western blot (Fig. 3e,f). These experiments suggest that the enhanced responsiveness to growth factors caused by Rab7a knockdown is a result of increased growth factor receptor density.

### Rab7a knockdown in mouse islets enhances IGF-1 induced cell signalling

**Figure 4:**
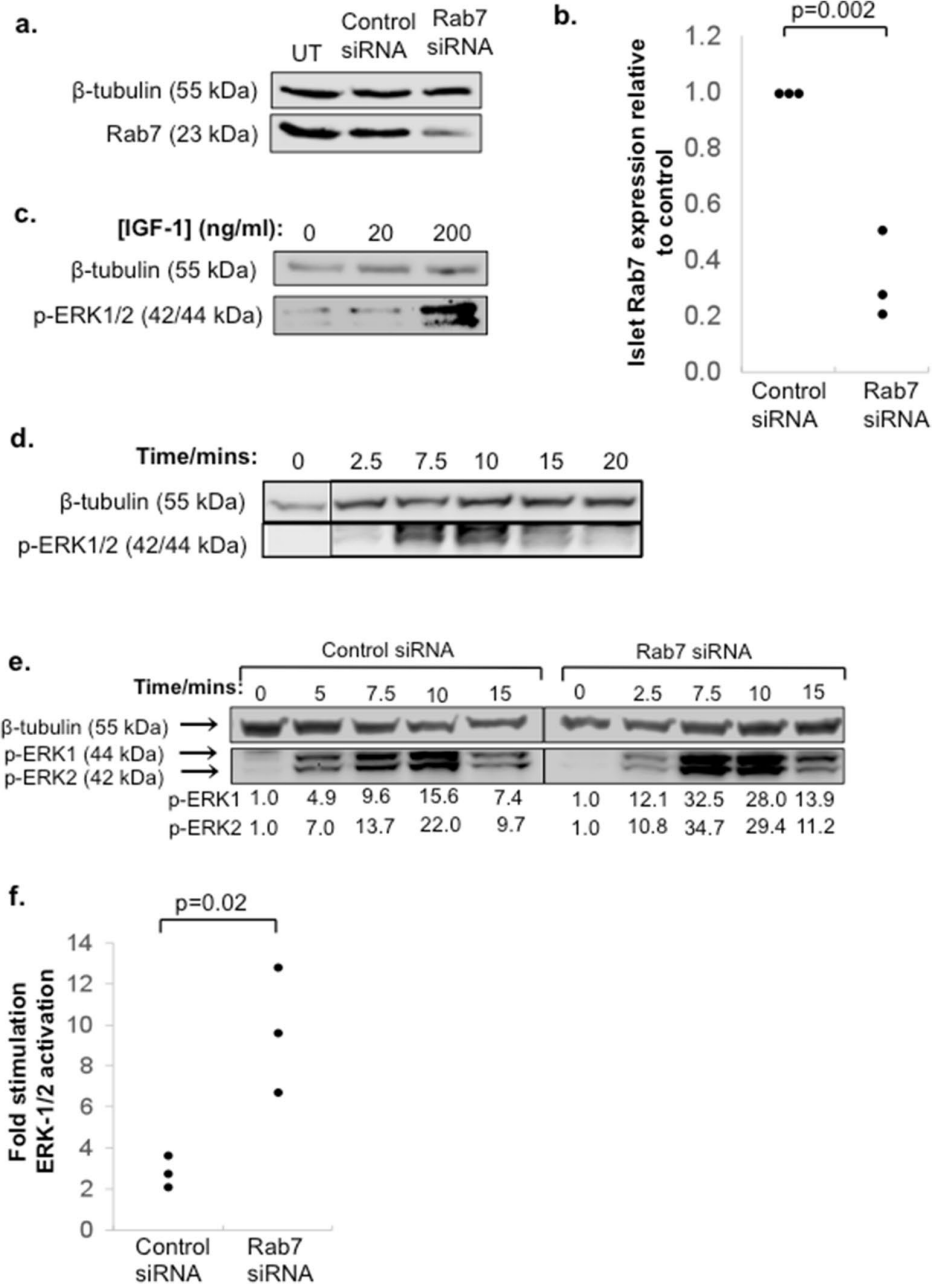
IGF-1 signalling is enhanced by Rab7a siRNA knockdown in pancreatic islets. (**a**) Islets were transfected with 150 nM Rab7a siRNA or negative control siRNA, or left untransfected (UT). After 3 days, the cells were analysed by Western blot for β-tubulin and Rab7a expression. A representative blot is shown in (**a**) and quantification of Rab7a expression standardised to β-tubulin is shown in (**b**), where the graph shows n = 3 data points from three independent experiments and the p value was calculated using Students’ t-test. (**c**, **d**) Show validation of the conditions required for ERK-1/2 phosphorylation in islets. Islets were culture overnight in low serum medium (0.5% FBS) then either stimulated with 0, 20 or 200 ng/ml IGF-1 for 7.5 min (**c**), or cultured with 200 ng/ml IGF-1 for the indicated times (**d**). Validation experiments in (**c**, **d**) were performed once to determine optimal conditions for subsequent experiments. In (**e**), islets were treated with 150 nM Rab7a siRNA or control siRNA, then cultured in low serum medium overnight before stimulation with 200 ng/ml IGF-1 for the indicated times. β-tubulin and p-ERK-1/2 expression were determined by Western blot. In (**e**) numbers under the blot indicate quantification of p-ERK-1/2 expression standardised to β-tubulin and relative to the untreated control, and the results are representative of two independent experiments. The graph in (**f**) is additional data from three further independent experiments showing the fold stimulation of phospho-ERK-1/2 after 7.5 min treatment with 200 ng/ml IGF-1 relative to unstimulated control (n = 3).

Having demonstrated that Rab7a enhances growth factor responsiveness in INS-1 cells, we then tested whether the same effect can be seen in primary mouse islets. Initial validation experiments demonstrated that pre-treatment with EDTA^28^ allowed us to reduce Rab7a expression up to or above the 70% threshold used in beta cells using 150 nM Rab7a siRNA (Fig. 4a,b). Validation of the optimal conditions for IGF-1 activation of ERK 1/2 signalling in islets showed that 200 ng/ml IGF-1 induced strong ERK-1/2 phosphorylation (Fig. 4c), and that peak activation in intact islets was observed at 7.5–10 min (Fig. 4d). Using these optimised conditions, we then tested the effect of Rab7a siRNA knockdown on IGF-1 signalling in islets. Islets transfected with control or Rab7a siRNA for 3 days were then cultured overnight in low serum medium before stimulation with 200 ng/ml IGF-1. As shown in Fig. 4e,f, ERK-1/2 phosphorylation was increased more than three-fold at 7.5 min, consistent with the effect seen previously in INS-1 cells. Rab7a inhibition therefore enhances growth factor signalling in primary mouse islets.

### Rab7a knockdown enhances growth factor induced islet survival

**Figure 5:**
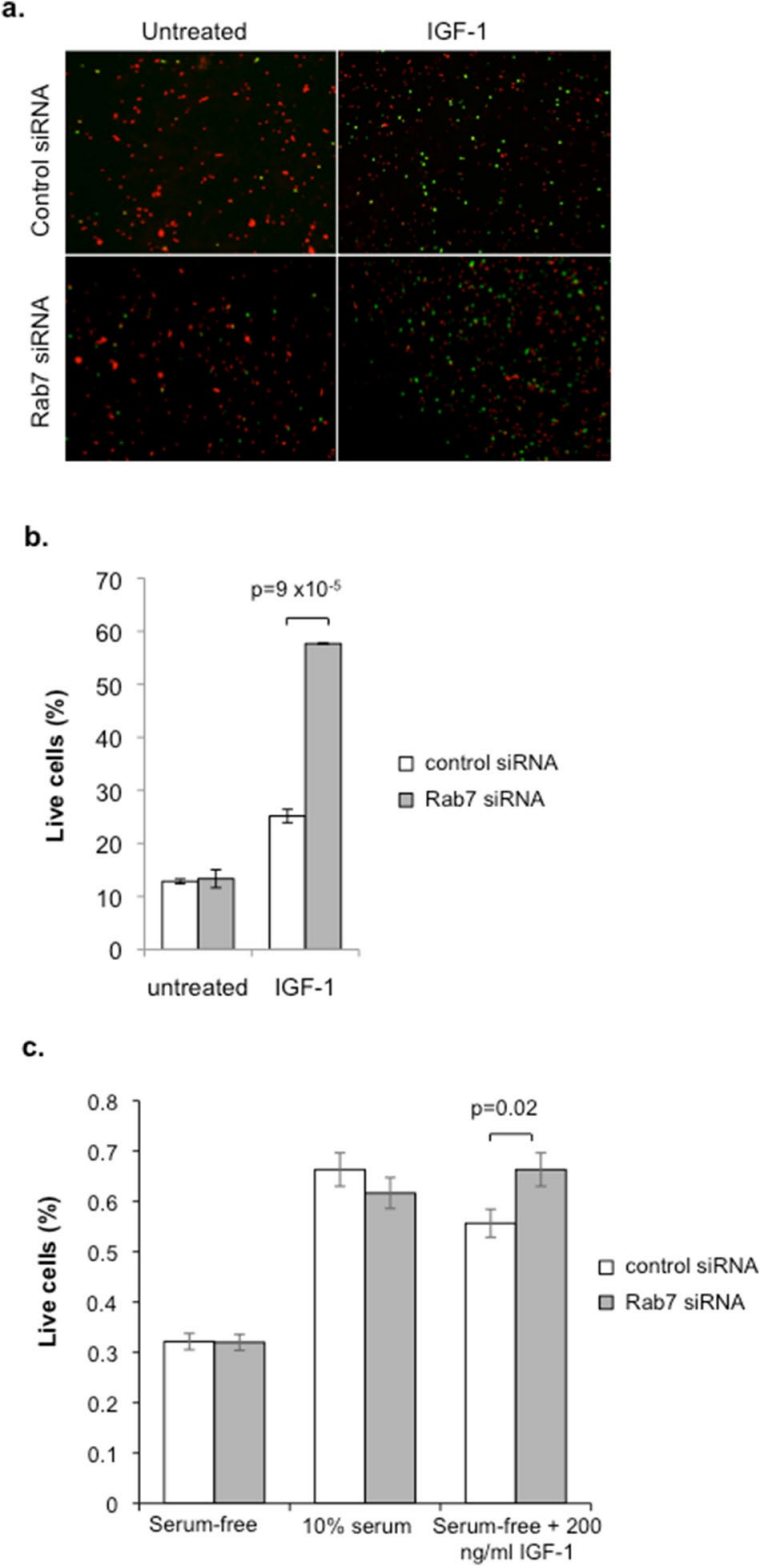
Pancreatic islet survival is enhanced by Rab7a siRNA knockdown. Islets were transfected with Rab7a siRNA or negative control siRNA for 3 days, then dispersed into single cells by Accutase treatment and seeded onto poly-lysine coated coverslips in complete medium. Islet cells were then serum starved (0% FBS) for 12 h, then either treated with or without 200 ng/ml IGF-1. After 2 days, cells were treated with calcein AM (green live stain) and ethidium homodimer-1 (red dead stain) and imaged. Representative images of the overlaid channels are shown (**a**), and (**b**) shows the mean % live cells for n = 3 independent experiments. (**c**) Similar experiments were also performed with intact islets (without Accutase treatment). As an additional control in this experiment, control- and Rab7a-siRNA treated cells did not undergo serum starvation and were instead maintained in 10% FBS. Graph shows mean % live cells (n = 3). Statistical analysis was performed using Students’ t-test.

Since the proliferative rate of beta cells in primary islets is very low, in order to test whether Rab7a knockdown affects growth factor responsiveness at the functional level we used a previously validated islet survival assay in which growth factor responsiveness is measured by the ability of growth factors to rescue islets from cell death induced by low serum conditions^26^. Islets were treated either with control- or Rab7a-siRNA as above, then dispersed into single cells and adhered to coated coverslips. Once adhered, the cells were serum starved for 12 h, then stimulated with or without IGF-1 for 2 days. Islet cell survival was then determined using a fluorescent live/dead assay. As expected, in control siRNA cells the presence of IGF-1 induced a two-fold increase in islet survival (Fig. 5a,b). However, this effect was dramatically enhanced in Rab7a siRNA treated cells, where a greater than 5-fold increase in islet survival was observed in response to IGF-1 treatment. Islet survival in response to growth factor treatment is therefore significantly greater in Rab7a siRNA treated cells compared to cells treated with control siRNA. This is consistent with the increase in growth factor signalling observed in Rab7a siRNA treated islets, and the enhanced growth in response to IGF-1 observed in INS-1 cells, and demonstrates that Rab7a inhibition is able to rescue beta cell loss resulting from low serum-stress, and enhances islet survival.

We also performed similar experiments in intact islets, rather than with single cells as before, and again saw enhanced responsiveness to IGF-1 in terms of increased survival in Rab7a siRNA treated islets (Fig. 5c). In these experiments we also performed additional controls, in which control- and Rab7a-siRNA treated islets did not undergo serum starvation and were instead maintained in standard 10% FBS conditions. Importantly, in the absence of serum-stress Rab7a knockdown did not significantly affect islet survival, suggesting that it is only under conditions of cellular stress that Rab7a has a significant affect in promoting islet survival.

### Rab7a siRNA knockdown protects against metabolic stress-induced autophagy and apoptosis.

The capacity for beta cells to withstand metabolic stress is thought to be critical in avoiding the development of type 2 diabetes. We therefore examined whether Rab7a attenuation can protect against metabolic stress. INS-1 cells were treated with varying concentrations of palmitic acid, a saturated fatty acid known to be lipotoxic to beta cells and often present at high levels in people with type 2 diabetes^37^. Palmitic acid treatment was seen to induce expression of both autophagy (expression of LC3-II) and apoptosis (caspase-3 cleavage) in INS-1 cells, as shown in Supplementary Fig. 1. In the absence of palmitic acid, Rab7a siRNA knockdown did not have a significant effect on either autophagy or apoptosis markers, as shown in Supplementary Fig. 2. This further supports our observations above that Rab7a only affects beta cells under conditions of cellular stress.

**Figure 6:**
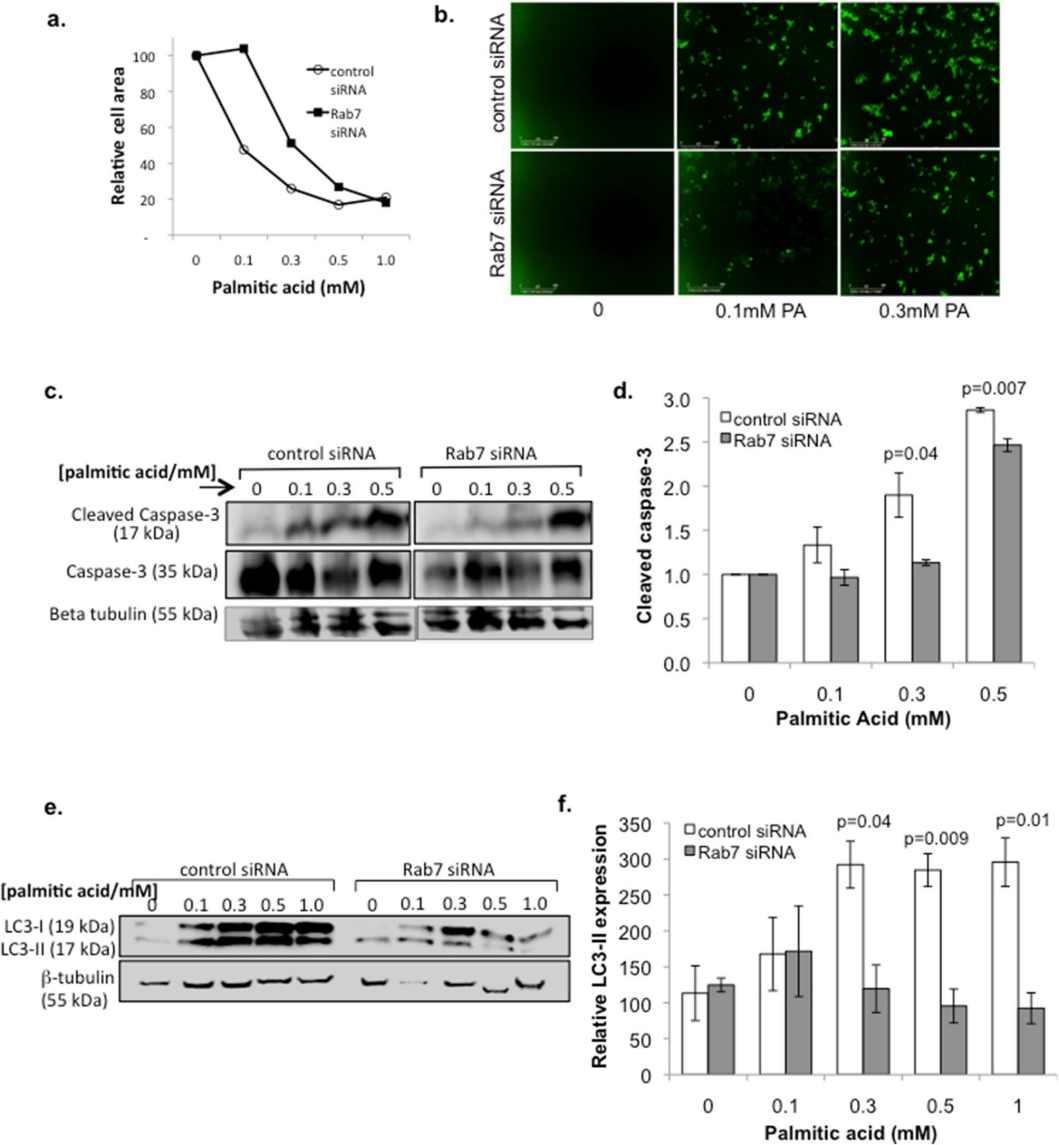
Rab7a attenuation protects against metabolic stress-induced beta cell death. INS-1 cells were treated with control- or Rab7a-siRNA for 3 days, then treated with the indicated concentration of palmitic acid (PA). (**a**) Cells were imaged using an IncucyteZoom system to determine cell area. (**b**) Representative images using 5 µM of Caspase-3/7 apoptosis reagent (Essen bioscience), which shows caspase-3/7 activation. Scale bar = 300 µm. (**c**) Cell lysates were prepared and analysed by western blot to quantify the amount of active (17 kDa) and inactive (35 kDa) Caspase-3. Representative blots are shown. (**d**) Relative mean expression of active caspase-3 is shown from three independent experiments. (**e**, **f**) Western blot analysis was used to determine expression of the autophagy marker LC3-I/II, with representative blots shown in (**e**), and relative mean expression of LC3-II from three independent experiments shown in (**f**). Values standardised to untreated control siRNA sample. Error bars indicate SEM, and Students’ t-test was used for statistical analysis.

We therefore tested the effect of palmitic acid-induced metabolic stress under conditions of Rab7a knockdown. As expected, in control siRNA-treated cells palmitic acid caused beta cell loss, with activation of both apoptosis (caspase-3/7 activation) and autophagy (expression of LC3-II) pathways, as shown in Fig. 6. However, Rab7a siRNA treatment inhibited this metabolic-stress induced beta cell loss and also inhibited the activation of both apoptotic and autophagic pathways, suggesting that attenuation of Rab7a promotes beta cell survival under conditions of metabolic stress.

## Discussion

In this study we have explored the novel hypothesis that regulation of growth factor receptor trafficking can be used to promote islet survival. We have shown for the first time that Rab7a regulates IGF-1 and HGF signalling, and that Rab7a inhibition enhances IGF-1R and c-Met receptor levels. Furthermore, our data demonstrates that inhibition of Rab7a can be used to enhance beta cell growth factor responsiveness and to promote islet survival under conditions of metabolic stress relevant to the conditions that occur in many people with type 2 diabetes.

It has been shown elsewhere that Rab7a is necessary for trafficking of cargo from late endosomes to lysosomes^36^. Although not directly tested here, it is therefore likely that Rab7a siRNA inhibition delays or prevents lysosomal degradation of IGF-1R and c-Met receptors, thereby causing the increased growth factor receptor density that we observe and conferring enhanced growth factor responsiveness. Delayed degradation could either promote receptor recycling via Rab4/Rab11 recycling pathways, resulting in reinsertion of receptors in the plasma membrane and increasing signalling at the cell surface; or, alternatively, could result in receptor accumulation in an endosomal compartment. Indeed, previous studies using Rab7a siRNA suggested that while Rab7a inhibition causes endosomal accumulation of EGFR, it also promotes receptor recycling of unliganded receptors to the cell surface via the slow peri-nuclear recycling pathway^38^. Another study has shown that Rab7a siRNA treatment decreases lysosomal trafficking of the Nrp1 receptor to enhance angiogenesis and, consistent with our experiments in beta cells, also suggests that Rab7a siRNA inhibition promotes growth factor responsiveness^23^. In the light of these findings, our data suggests that Rab7a siRNA treatment enhances islet survival by increasing pro-survival growth factor signalling from either an endosomal compartment, or by promoting receptor recycling to the cell surface.

Interestingly, the experiments used in our study involve either serum-starvation prior to growth factor treatment, or mimic the metabolic stress conditions associated with type 2 diabetes using high levels of palmitic acid. It is known that serum-starvation and other conditions of cellular stress result in the activation of a co-ordinated programme of stress responses, including both autophagic degradation of cytoplasmic components and the endocytic degradation of cell surface receptors^39^. These two pathways converge at the point of lysosomal fusion^40^. Rab7a is known to regulate both autophagy and the endocytic pathways, and Rab7a is itself activated by cell stress^20,24,41^. This has important implications since in type 2 diabetes islet beta cells are subject to stressful conditions due to high levels of fatty acids and glucose, as well as endoplasmic reticulum stress due to high secretory activity as a result of increased insulin demand^42^. Islet transcriptomic studies have previously shown that IGF-1R is downregulated by both metabolic and inflammatory cytokine stress^43,44^. Therapeutic inhibition of Rab7a may therefore be particularly effective in protecting islet beta cells under the conditions of metabolic stress that exist in many diabetes patients, since in other cell types and under normal conditions the majority of endosomal cargo is recycled to the plasma membrane and lysosomal degradation of growth factor receptors is low^40^. Consistent with this, our data suggests that the effects of Rab7a inhibition in non-stressed cells are very limited, conferring specificity to struggling beta cells. The lack of enhanced growth factor responsiveness in unstressed cells also suggests that Rab7a inhibition is effective in preventing the degradation of growth factor receptors during cellular stress, with positive effects on islet survival, but does not promote a potentially dangerous accumulation of growth factor receptors under ‘normal’ conditions. The endocytic pathway is highly conserved across species. Therapeutic Rab7a inhibition to maintain growth factor receptor density and subsequent growth factor responsiveness to protect islets may therefore be an effective approach to enable beta cells to survive the metabolic stress they are subjected to during diabetes pathogenesis. Targeting Rab7a therefore represents a novel potential therapeutic approach to promoting islet survival in type 2 diabetes patients.

## Supplementary information

Supplementary Figures.
