# Correction: Modulation of Rab7a-mediated growth factor receptor trafficking inhibits islet beta cell apoptosis and autophagy under conditions of metabolic stress

**DOI:** 10.1038/s41598-025-20209-0

**Published:** 2025-09-19

**Authors:** Nirun V. Hewawasam, Fadel Lhaf, Henry A. Taylor, Katrina Viloria, Amazon Austin, Aileen King, Peter Jones, Lucy Jones, Mark D. Turner, Natasha J. Hill

**Affiliations:** 1 Department of Biomolecular Sciences, Kingston University, Penrhyn Road, Kingston-Upon-Thames, KT1 2EE UK; 2 Department of Diabetes, Kings College London, London, UK; 3 School of Science and Technology, Nottingham Trent University, Nottingham, UK

Correction to: *Scientific Reports* 10.1038/s41598-020-72939-y, published online 25 September 2020

This Article contains an error in Figure 6b, where the image for active Caspase-3/7 in control siRNA cells treated with 0.1mM palmitic acid (PA) was a duplication of the image for Rab7 siRNA cells treated with 0.3mM PA.

The correct Figure 6 and its accompanying legend appear below.

**Fig. 6:**
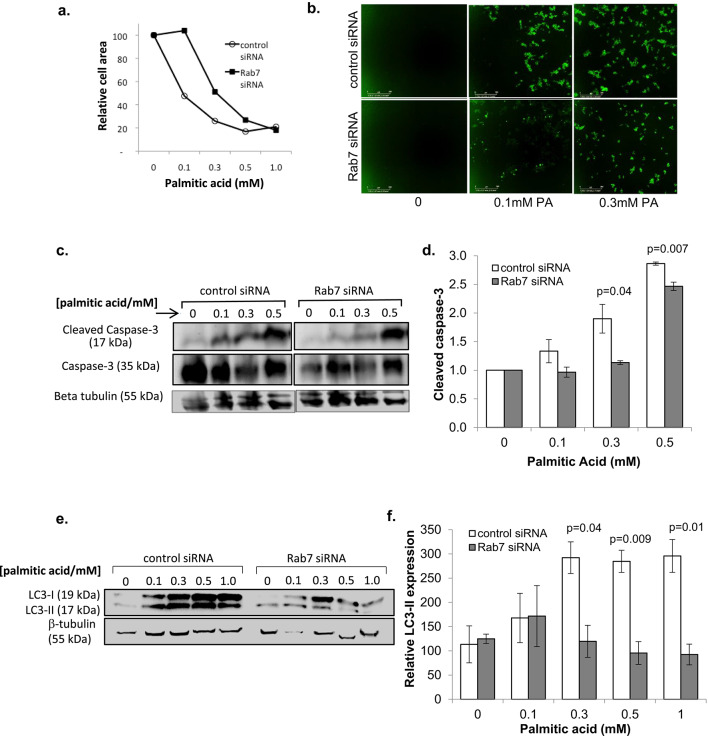
Rab7a attenuation protects against metabolic stress-induced beta cell death. INS-1 cells were treated with control- or Rab7a-siRNA for 3 days, then treated with the indicated concentration of palmitic acid (PA). (**a**) Cells were imaged using an IncucyteZoom system to determine cell area. (**b**) Representative images using 5 µM of Caspase-3/7 apoptosis reagent (Essen bioscience), which shows caspase-3/7 activation. Scale bar = 300 µm. (**c**) Cell lysates were prepared and analysed by western blot to quantify the amount of active (17 kDa) and inactive (35 kDa) Caspase-3. Representative blots are shown. (**d**) Relative mean expression of active caspase-3 is shown from three independent experiments. (**e**, **f**) Western blot analysis was used to determine expression of the autophagy marker LC3-I/II, with representative blots shown in (**e**), and relative mean expression of LC3-II from three independent experiments shown in (**f**). Values standardised to untreated control siRNA sample. Error bars indicate SEM, and Students’ t-test was used for statistical analysis.

