# Supplementary material for: Modulation of Rab7a-mediated growth factor receptor trafficking inhibits islet beta cell apoptosis and autophagy under conditions of metabolic stress
Source: Sci Rep. 2020 Sep 25;10:15741. doi: 10.1038/s41598-020-72939-y (PMC7519639; doi:10.1038/s41598-020-72939-y)
Supplement: Supplementary file 1 — Supplementary Figures. [file 41598_2020_72939_MOESM1_ESM.pdf]

Modulation of Rab7a-mediated growth factor receptor trafficking inhibits islet beta cell apoptosis and autophagy under conditions of metabolic stress.

Nirun V Hewawasam[1¶], Fadel Lhaf[1¶], Henry A Taylor[1], Katrina Viloria[1], Amazon Austin[2], Aileen King[2], Peter Jones[2], Lucy Jones[1], Mark D Turner[3] and Natasha J Hill[1*]

Supplementary Figure 1.

a.

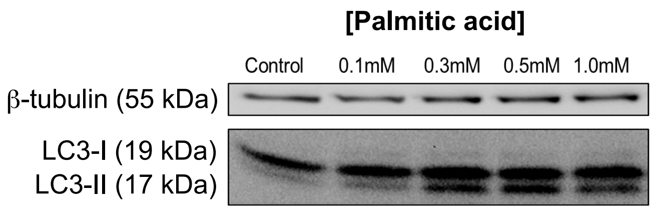

b.

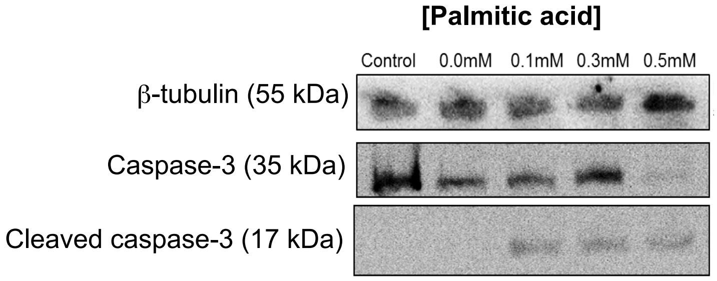

Supplementary Figure 1: Palmitic acid induces activation of both autophagy and apoptosis pathways. INS-1 cells were seeded at 1X10'5 cells/ml in 24 well plates in standard 10% FBS medium and allowed to adhere overnight before treatment with the indicated concentration of palmitic acid. Lysates were then analysed by western blot using antibodies to the autophagy marker LC3-I/II (a) and the apoptosis marker Caspase-3 (b). The active cleaved form of Caspase-3 was only detected following treatment with at least 0.1mM palmitic acid.

Supplementary Figure 2.

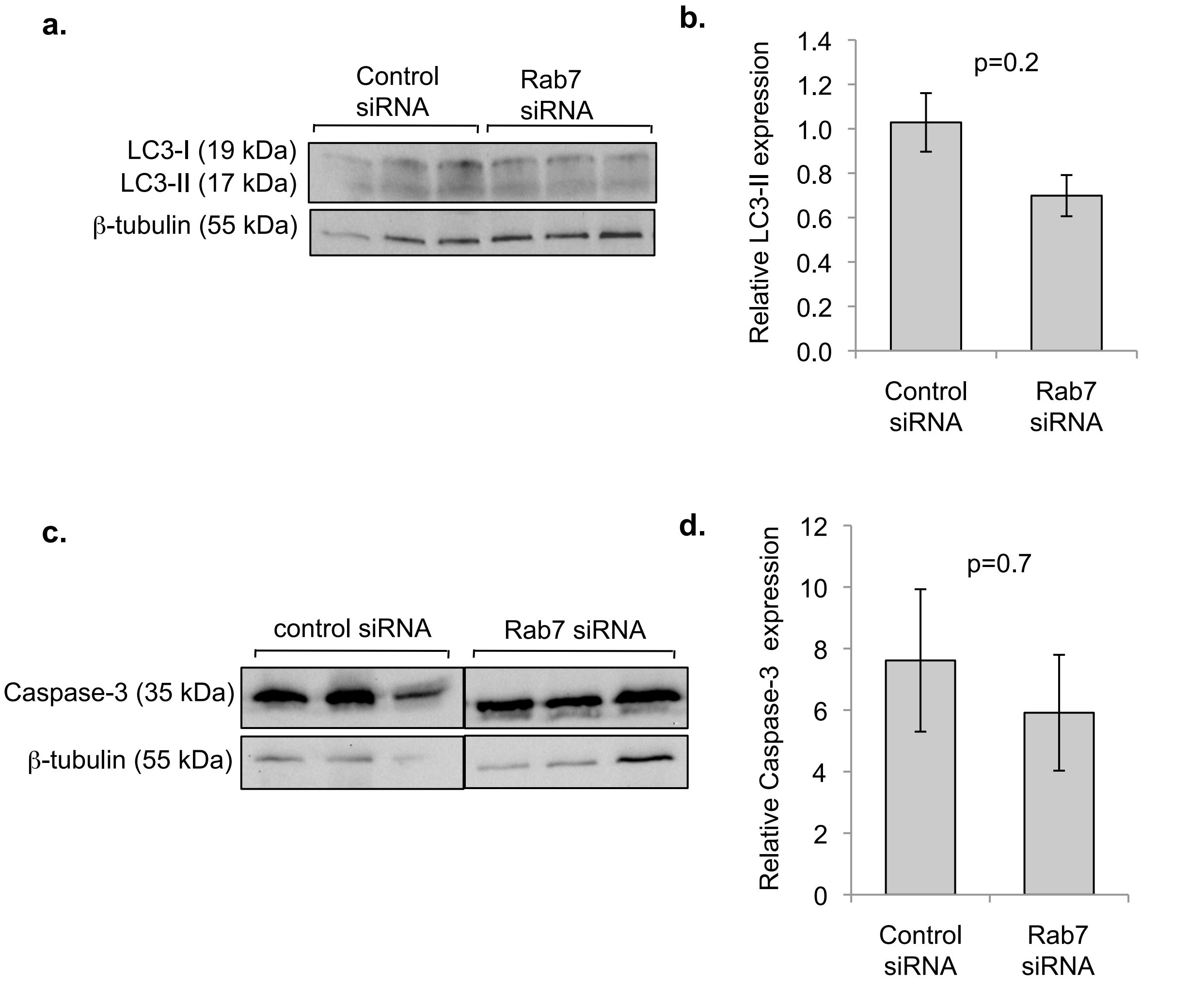

Supplementary Figure 2: Rab7 siRNA knockdown does not affect expression of autophagy and apoptosis markers in unstressed conditions. INS-1 cells were seeded at 1X10’5 cells/ml in 24 well plates and transfected with control- or Rab7a-siRNA for 72 hours in standard 10% FBS medium, before preparing lysates for western blot using antibodies to the autophagy marker LC3 (a&b) and the apoptosis marker Caspase-3 (c&d). No cleaved caspase-3 was observed in the absence of palmitic acid. Graphs in B&D shown mean relative expression standardised to β tubulin, +/- SEM, from 3 independent experiments.

Uncropped blots, Hewawasam et al.

Figure 1

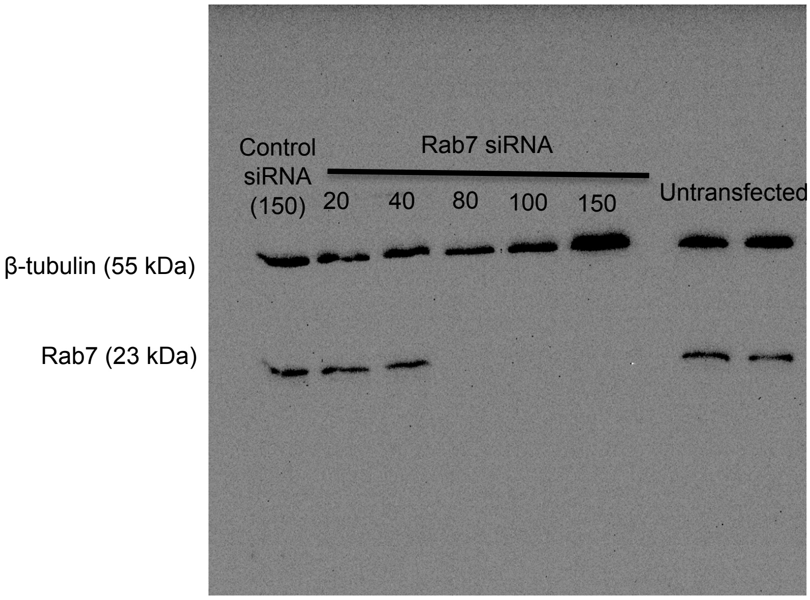
Control siRNA (150)
Rab7 siRNA
20
40
80
100
150
Untransfected
β-tubulin (55 kDa)
Rab7 (23 kDa)

Figure 2

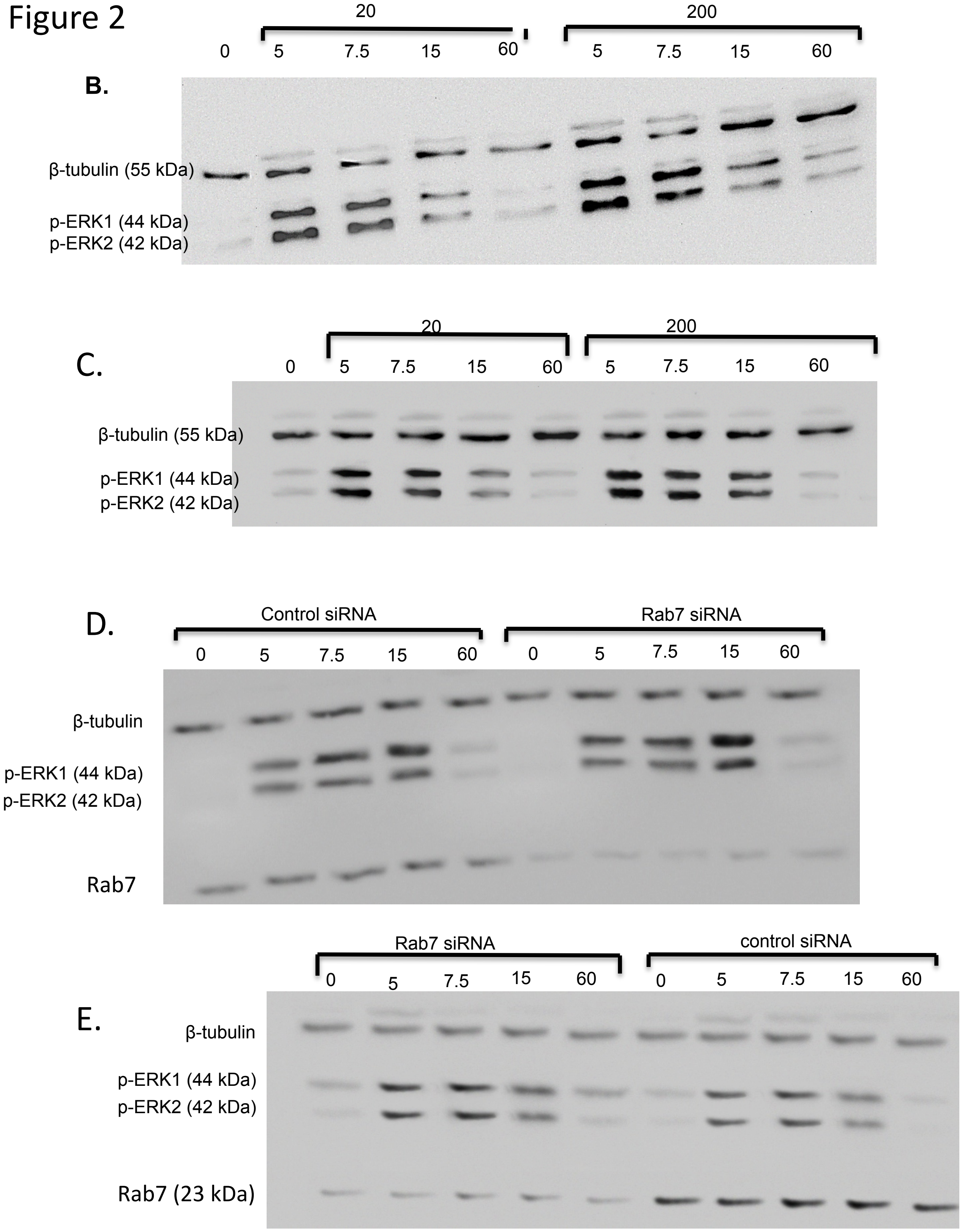

B.
20
200
0
5
7.5
15
60
β-tubulin (55 kDa)
p-ERK1 (44 kDa)
p-ERK2 (42 kDa)
C.
20
200
0
5
7.5
15
60
β-tubulin (55 kDa)
p-ERK1 (44 kDa)
p-ERK2 (42 kDa)
D.
Control siRNA
Rab7 siRNA
0
5
7.5
15
60
β-tubulin
p-ERK1 (44 kDa)
p-ERK2 (42 kDa)
Rab7
E.
Rab7 siRNA
control siRNA
0
5
7.5
15
60
β-tubulin
p-ERK1 (44 kDa)
p-ERK2 (42 kDa)
Rab7 (23 kDa)

Figure 3

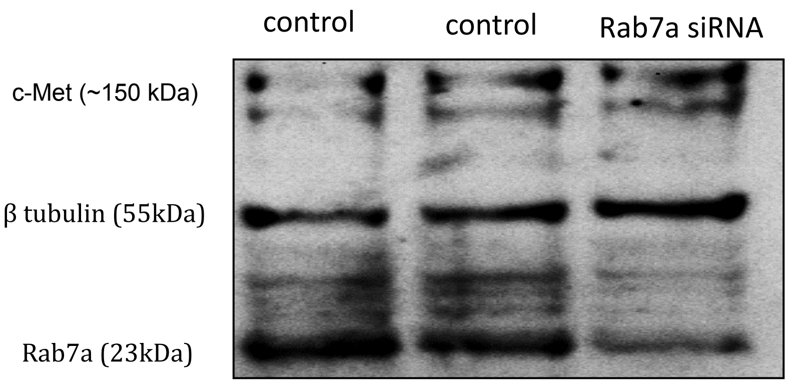
control
control
Rab7a siRNA
c-Met (~150 kDa)
β tubulin (55kDa)
Rab7a (23kDa)

Figure 4

A.

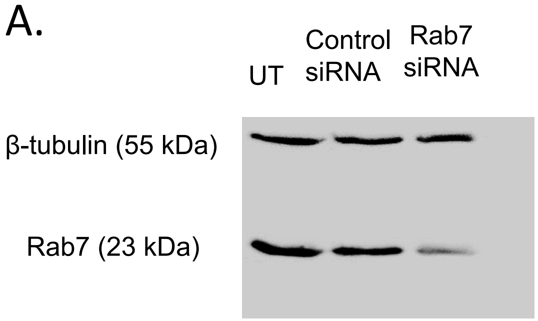

C.

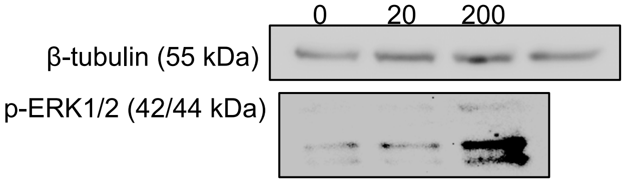

D.

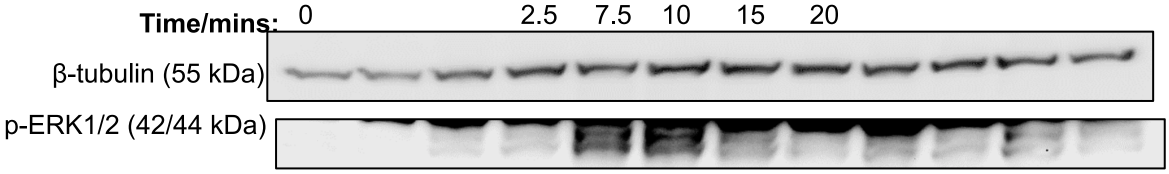

Fully uncropped blots for validation data in (C&D) to determine optimum concentration and time of stimulation could not be located.

E.

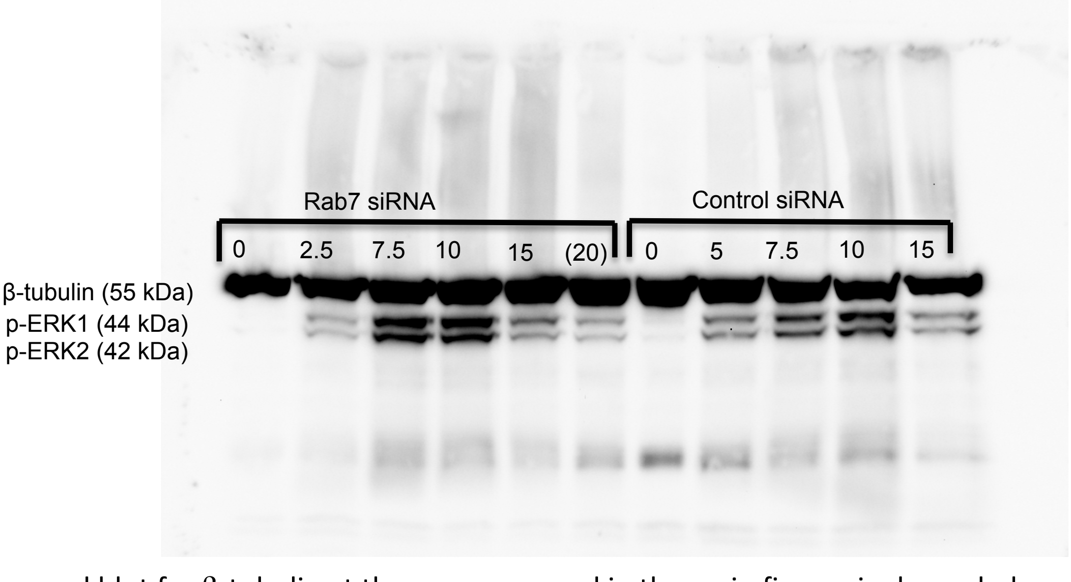

Uncropped blot for β-tubulin at the exposure used in the main figures is shown below *(fully uncropped blot at this exposure level not available)*

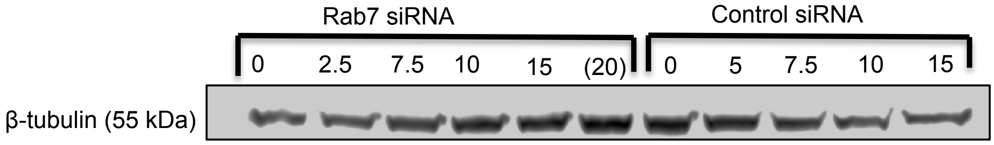

NB The lanes in (E) have been re-ordered in the final figure for consistency.

Figure 6

C.

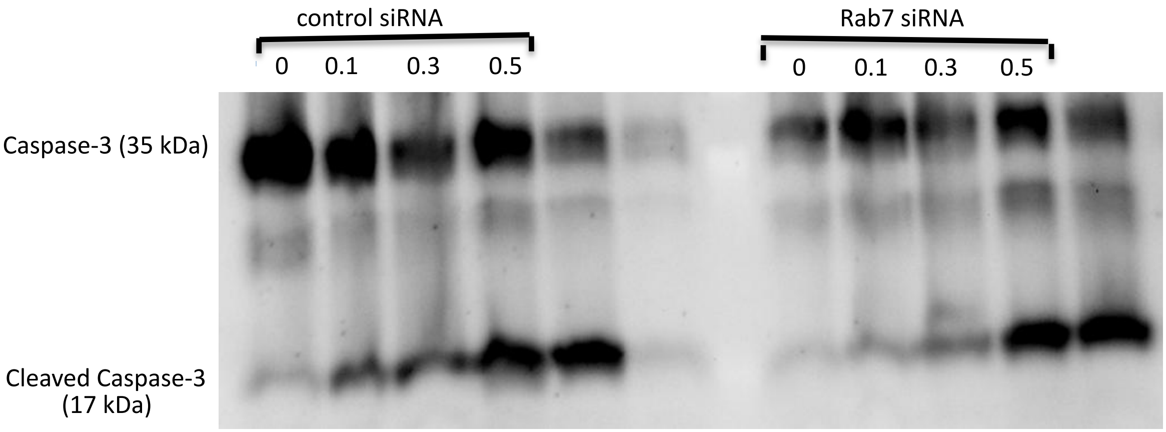

Fully uncropped image showing tubulin bands could not be located.

E.

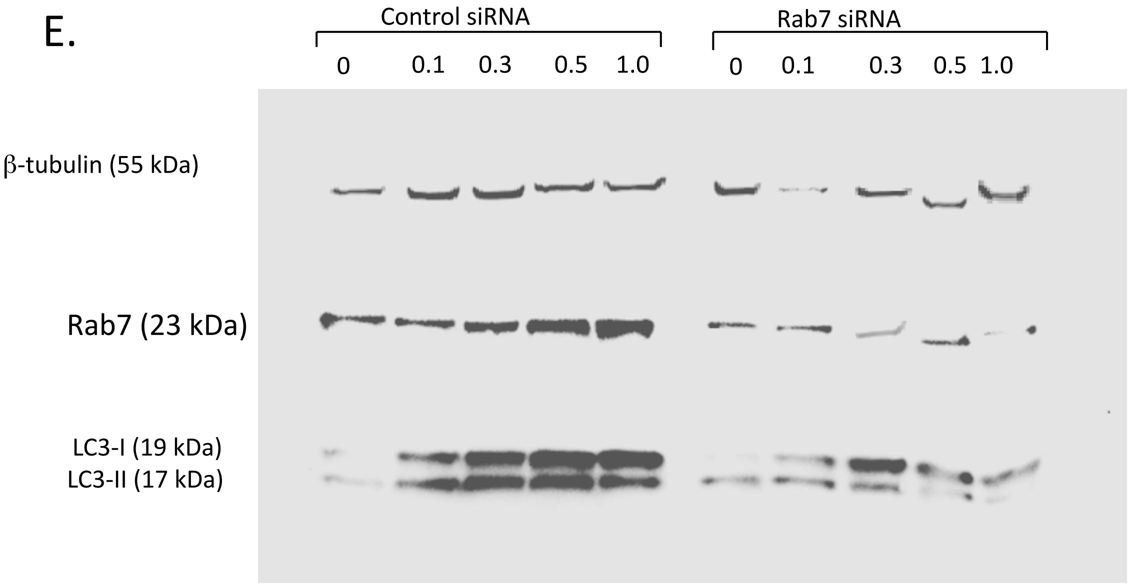

Supplementary Figure 1

a.

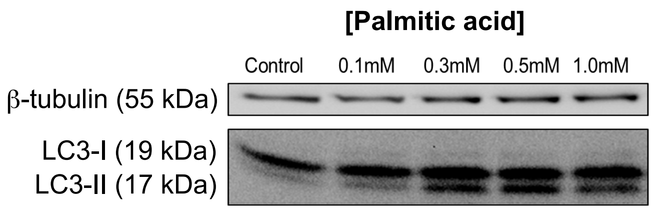

b.

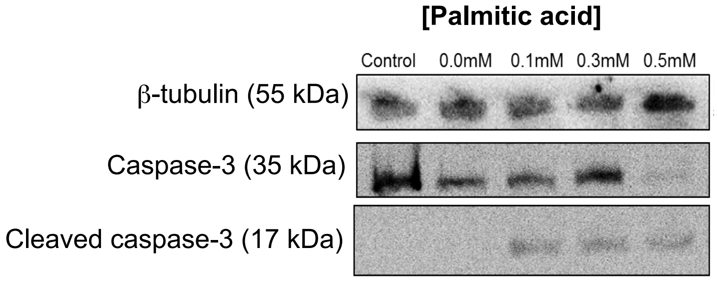

We regret that uncropped blots could not be located for this figure.

Supplementary Figure 2

A.

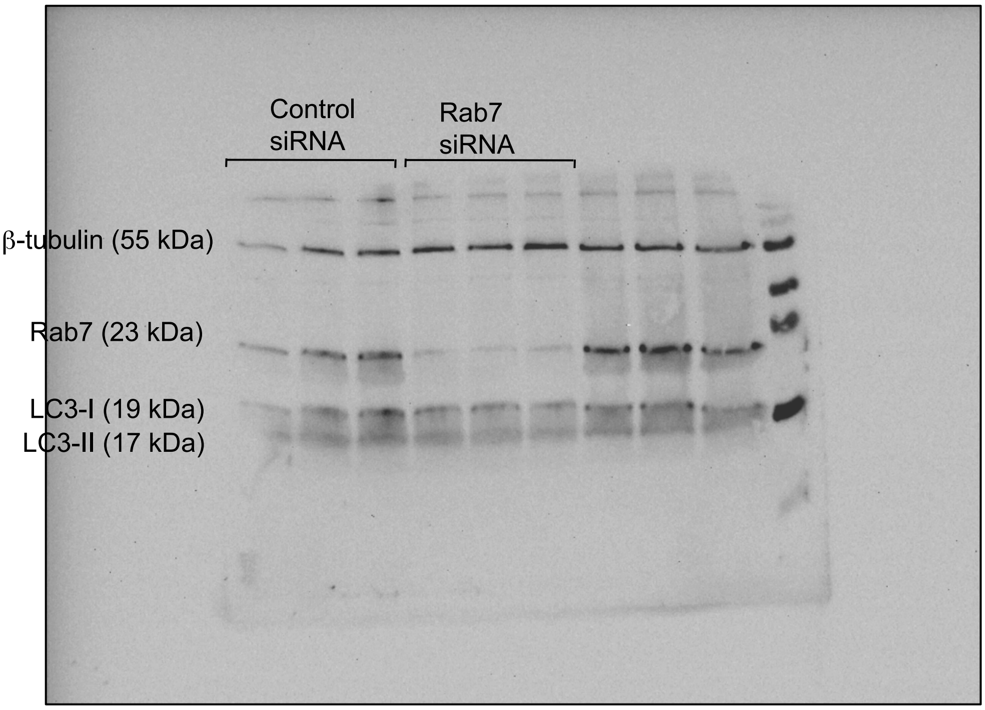

C.

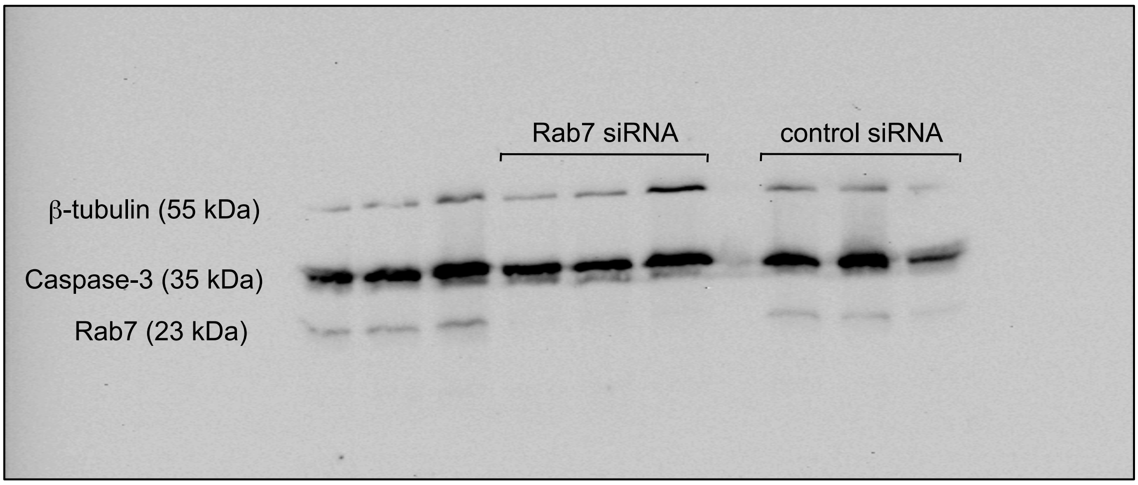

NB The lanes have been re-ordered in the final figure for consistency.
